# Association analysis of complex diseases using triads, parent-child dyads and singleton monads

**DOI:** 10.1186/1471-2156-14-78

**Published:** 2013-09-04

**Authors:** Ruzong Fan, Annie Lee, Zhaohui Lu, Aiyi Liu, James F Troendle, James L Mills

**Affiliations:** 1 Biostatistics and Bioinformatics Branch, Division of Intramural Population Health Research, Eunice Kennedy Shriver National Institute of Child Health and Human Development, National Institutes of Health, 6100 Executive Blvd, MSC 7510, Rockville, MD 20852, USA; 2 Department of Biostatistics, Mailman School of Public Health, 722 West 168th street, 6th floor, Columbia University, New York, NY 10032, USA; 3 Office of Biostatistics Research, Division of Cardiovascular Sciences, National Heart, Lung, and Blood Institute, National Institutes of Health, Bld. RKL2, Room 9196, Bethesda, MD 20892, USA; 4 Epidemiology Branch, Division of Intramural Population Health Research, Eunice Kennedy Shriver National Institute of Child Health and Human Development, National Institutes of Health, 6100 Executive Blvd, MSC 7510, Rockville, MD 20852, USA

**Keywords:** Association mapping of complex diseases, Likelihood ratio tests, Transmission disequilibrium tests

## Abstract

**Background:**

Triad families are routinely used to test association between genetic variants and complex diseases. Triad studies are important and popular since they are robust in terms of being less prone to false positives due to population structure. In practice, one may collect not only complete triads, but also incomplete families such as dyads (affected child with one parent) and singleton monads (affected child without parents). Since there is a lack of convenient algorithms and software to analyze the incomplete data, dyads and monads are usually discarded. This may lead to loss of power and insufficient utilization of genetic information in a study.

**Results:**

We develop likelihood-based statistical models and likelihood ratio tests to test for association between complex diseases and genetic markers by using combinations of full triads, parent-child dyads, and affected singleton monads for a unified analysis. A likelihood is calculated directly to facilitate the data analysis without imputation and to avoid computational complexity. This makes it easy to implement the models and to explain the results.

**Conclusion:**

By simulation studies, we show that the proposed models and tests are very robust in terms of accurately controlling type I error evaluations, and are powerful by empirical power evaluations. The methods are applied to test for association between transforming growth factor alpha (*TGFA*) gene and cleft palate in an Irish study.

## Background

In family-based studies, one might collect triads, sib-ships, parent-child dyads, general pedigrees or some combinations. In modern times, large multi-generation pedigrees are not common, and small nuclear families are more practical to collect. In our birth defects studies, almost all families contain only a single affected child with or without parents. They are basically triad families allowing for missing parents [1]. In family association studies, triad families are routinely used to test association between genetic variants and complex diseases. Triad studies are important and popular since they are robust in terms of being less prone to false positive results due to population structure [2,3]. In particular, triad studies are advantageous over case control designs which are prone to spurious association due to population stratification.

In practice, one may collect not only complete triads, but also incomplete families such as dyads (affected child with one parent) and singleton monads (affected child without parents). Here the terminology of dyads and monads are taken from Weinberg [4]. Since there is a lack of convenient algorithms and software to analyze the incomplete data, dyads and monads are usually discarded. This may lead to loss of power and insufficient utilization of genetic information in a study. For instance, dyads and monads were not used in the analysis of family data in the Irish oral clefts study [1]. This study contained about 75% triads and 25% parent-child dyads in addition to some affected monads. Only triads were used in an analysis of transmission disequilibrium tests (TDT) [1]. The reason that parent-child dyads and singleton monads were not used in the analysis is that there is no readily available software to analyze the combinations of triads, dyads, and monads, although statistical models are proposed in the literature to analyze family data jointly [5-9]. Intuitively, analyzing combined data should improve the power compared with the methods which use triads only, and should be more robust since more data are added to the analysis. Therefore, it is important and interesting to develop statistical models and related software to analyze the combined data of triads, dyads, and monads.

Triad studies are popular and important because the triad families are relatively easy to collect. More importantly, the results of triad studies are robust in terms of being less prone to false positives due to population stratification. To analyze triad data, TDT analysis is usually performed [3]. To use both triads and dyads for a combined analysis, Sun et al. [10] proposed a score test to more sufficiently use the data information. To use more data in the analysis, a likelihood-based approach was developed to handle missing data by imputation. For instance, an EM algorithm was used to recover the information contained in dyads and monads in Epstein et al. [11] and Weinberg [4]. Specifically, Epstein et al. [11] proposed a likelihood based approach to analyze the combinations of the family data handling missing parent data by imputation. The imputation arguments are based on similar derivation of Schaid and Sommer [12], p1119, right column. In addition, Nagelkerke et al. [13] used an approximate analysis of logistic regression. The joint analysis and design of family data has received extensive research in the last decade [14-17]. Some efforts have been made to implement the statistical models to software [18]. However, it is desirable to build statistical models which can be easily implemented to handle specific data such as the family data of the Irish oral clefts study, and to explain the results easily.

In this paper, we develop likelihood-based statistical methods to test for association between complex diseases and genetic markers by using combinations of full triads, parent-child dyads, and affected singleton monads for a unified analysis. Our research interest is stimulated by our oral clefts study [1]. We assume that the data are ascertained through the affected cases, i.e., the triads and parent-child dyads are ascertained through the affected child, and the affected monads are ascertained via themselves. Some studies use conditional likelihood given the parent mating type, which is not appropriate for our birth defects study since the data are ascertained through the affected cases [12].

Assume that we have a di-allelic candidate gene locus such as a single nucleotide polymorphism (SNP). We derive the conditional probabilities of triad, dyad, and monad genotypes given the sampling scheme that the data are ascertained through the affected cases. A conditional likelihood is then constructed directly; the likelihood is calculated without imputation; and analytical formulae are provided for parameter estimations, which are presented in Appendix A of Additional file 1. Based on the likelihood, likelihood ratio tests (LRT) are performed to test for association between complex diseases and genetic markers. To evaluate the performance of the proposed models and tests in terms of robustness and power, extensive simulation studies are carried out to calculate the empirical type I error rates and powers. From simulation results, we show that the proposed methods are very robust in terms of correct empirical type I error rates, and the methods are powerful. The methods are applied to test for association between the transforming growth factor alpha (*TGFA*) gene and cleft palate in the Irish study [1]. The proposed methods are programmed by the statistical package R to facilitate the data analysis.

## Results

Extensive simulations are carried out to evaluate the performance of the proposed models and tests. The robustness of the test statistics is evaluated by empirical type I rates. The power performance is evaluated by empirical power analysis. The simulation strategy is presented in the **Methods** section.

### Empirical type I error rates

The results of empirical type I error rates are presented in Table 1. For each entry of Table 1, we simulate 100,000 datasets under the null hypothesis *H*_0_:*ψ*_1_=*ψ*_2_=1, where *ψ*_1_ and *ψ*_2_ are relative risks defined in the **Methods** section. Each dataset contains *s*=50 affected monads and *n*=100,200, or 500 triads. For each of the three cases, *m*=0 or *m*=0.25*n* parent-child dyads are simulated in the dataset. For instance, *m*=0 or *m*=25 parent-child dyads are generated in the dataset when the number of affected monads is *s*=50 and the triad number is *n*=100. An empirical test statistic is calculated for each dataset. The empirical type I error rates at nominal levels *α*=0.05 and *α*=0.01 are reported in Table 1 which represent the proportions of false positives in the 100,000 replicates, that is, proportions of test values which exceed the 95-th and 99-th percentiles of the $\chi_{2}^{2}$ (for *Unr* model) or $\chi_{1}^{2}$ (for *Dom*, *Rec*, *Mult*, and *Add* models) distributions, respectively.

**Table 1:** Empirical type I error rates at 0.05 and 0.01 nominal significance levels of the proposed tests

**Nominal**	**Sample size**			**p**	**Model**				
**Level *α***	**s**	**n**	**m**		**Unr**	**Dom**	**Rec**	**Mult**	**Add**
				0.5	0.04989	0.04985	0.04867	0.05028	0.05138
			25	0.2	0.04152	0.05057	0.04240	0.05214	0.05364
0.05	50	100		0.1	0.03517	0.05006	0.02672	0.04979	0.05230
				0.05	0.05327	0.05093	0.05038	0.05138	0.05685
				0.5	0.04921	0.05055	0.05135	0.05149	0.05330
			0	0.2	0.03999	0.05073	0.03637	0.05156	0.05222
				0.1	0.03534	0.05088	0.02611	0.05249	0.05199
				0.05	0.05944	0.04916	0.06505	0.05230	0.05609
				0.5	0.00907	0.01039	0.00973	0.00972	0.01031
			25	0.2	0.00759	0.01031	0.00568	0.01043	0.01117
0.01	50	100		0.1	0.00699	0.01011	0.00504	0.01054	0.00936
				0.05	0.01010	0.01072	0.00966	0.01021	0.01200
				0.5	0.00831	0.0098	0.01008	0.00998	0.01043
			0	0.2	0.00723	0.01075	0.00489	0.01054	0.01097
				0.1	0.00676	0.01065	0.00477	0.01040	0.01014
				0.05	0.01054	0.01054	0.01204	0.01104	0.01163
				0.5	0.04974	0.05003	0.05093	0.04951	0.05178
			50	0.2	0.04641	0.04976	0.05019	0.05005	0.05201
0.05	50	200		0.1	0.03539	0.04977	0.02236	0.04996	0.05236
				0.05	0.04095	0.05142	0.03730	0.04990	0.05288
				0.5	0.05121	0.05027	0.05121	0.05039	0.05098
			0	0.2	0.04360	0.05128	0.04991	0.05210	0.05249
				0.1	0.03503	0.05018	0.02303	0.05146	0.05108
				0.05	0.04521	0.05156	0.04011	0.05423	0.04997
				0.5	0.01042	0.00967	0.01010	0.00961	0.01050
			50	0.2	0.00872	0.00962	0.00914	0.01020	0.01101
0.01	50	200		0.1	0.00642	0.00986	0.00430	0.01056	0.01043
				0.05	0.00769	0.01125	0.00705	0.01091	0.01133
				0.5	0.00987	0.00976	0.00987	0.00991	0.01002
			0	0.2	0.00799	0.01030	0.00828	0.01059	0.01062
				0.1	0.00629	0.01012	0.00443	0.00994	0.01089
				0.05	0.00900	0.01027	0.00769	0.01076	0.00924
				0.5	0.05032	0.05009	0.05048	0.04870	0.04895
			125	0.2	0.05091	0.05029	0.05025	0.04965	0.05111
0.05	50	500		0.1	0.04178	0.04998	0.04298	0.05023	0.05021
				0.05	0.03380	0.05043	0.02511	0.05020	0.05081
				0.5	0.04957	0.05049	0.05056	0.05150	0.05048
			0	0.2	0.05071	0.04963	0.0522	0.04954	0.05084
				0.1	0.03980	0.05037	0.03109	0.04936	0.05067
				0.05	0.03491	0.05012	0.02749	0.05031	0.05107
				0.5	0.01032	0.00957	0.01016	0.00963	0.00928
			125	0.2	0.01006	0.01045	0.01031	0.01040	0.01062
0.01	50	500		0.1	0.00707	0.0098	0.00502	0.01051	0.01008
				0.05	0.00640	0.00996	0.00473	0.00979	0.01077
				0.5	0.00971	0.01057	0.00974	0.01024	0.00998
			0	0.2	0.00978	0.00999	0.01081	0.00955	0.01010
				0.1	0.00725	0.01007	0.00484	0.00974	0.01078
				0.05	0.00677	0.00990	0.00525	0.01010	0.01044

Encouragingly, the empirical type I error rates were all around or below the nominal levels 0.05 and 0.01, except two entries 0.05944 and 0.06505 of unrestricted (*Unr*) and recessive (*Rec*) models when the allele frequency *p*=0.05, triad size *n*=100, monad size *s*=50, and dyad size *m*=0. Hence, the proposed test statistics are very robust. Table 1 exhibits an interesting trend: the type I error rates of the dominant (*Dom*), multiplicative (*Mult*), and additive (*Add*) columns are not affected by the allele frequency *p* but the error rates of the other two columns for *Unr* and *Rec* are generally getting smaller when *p* decreases except when *p*=0.05 and the sample sizes are small. This shows that the models of *Unr* and *Rec* are getting more conservative when the allele frequency *p* is getting smaller except when *p*=0.05 and the sample sizes are small.

### Power analysis

Power analysis is performed to calculate empirical power levels for various scenarios. The results are presented in Table 2 and Table 3. To make our results comparable with those of Table 3, Troendle et al. [19], we use the same parameters in our simulation. Again, we simulate 100,000 datasets for each entry of Table 2 and Table 3. The empirical power levels at nominal levels *α*=0.05 are reported. They show four notable results. First, the power levels in Table 2 and Table 3 for most entries are higher than those corresponding entries of Table three, Troendle et al. [19]; for a few entries, they are slightly lower. Hence, the proposed models are reasonably powerful. Second, the power levels obtained by using combinations of both triads and parent-child dyads are higher than those obtained by using triads only. Tests using combinations of triads, parent-child dyads and singleton monads provide the highest power levels. Thus, it is advantageous to use more data in the analysis. Third, the power levels are the highest when the disease models are correctly specified (the results on the diagonals marked by boldface in Table 2 and Table 3). However, if dominant disease is misspecified as recessive or vice versa, it leads to powerless tests. If dominant disease or recessive disease is misspecified as unrestricted or multiplicative, the power loss is less severe. Fourth, TDT and *z*_*com*_ suffer severe power loss compared with the correctly specified LRT statistics. Overall, the power levels of TDT and *z*_*com*_ are lower than the proposed LRT. Hence, the proposed parametric models can be very useful in mapping disease genes.

**Table 2:** **Power performance of the proposed tests at 0.05 nominal significance level using the parameters in Table three, Troendle et al. [**[19]**] and *n = 100***

**Sample size**			**p**	**Disease model**		**Model**					**TDT**	***z***_***com***_
**s**	**n**	**m**		**Model**	**(*ψ***_**1**_**,*ψ***_**2**_**)**	**Dom**	**Rec**	**Mult**	**Unr**	**Add**		
				**Dom**	(2.6, 2.6)	**0.91066**	0.06000	0.50500	0.84541	0.70714		0.31163
		25	0.5	**Rec**	(1.0, 2.2)	0.04523	**0.90067**	0.70559	0.83608	0.57124		0.47878
				**Mult**	(1.8, 3.24)	0.48646	0.68023	**0.88068**	0.80862	0.87348		0.65977
0	100			**Unr**	(0.65, 1.54)	0.32262	0.93172	0.34347	**0.92475**	0.17819		0.22527
				**Dom**	(2.6, 2.6)	**0.84710**	0.05676 ^∗^	0.44357	0.76226	0.63856	0.44186	
		0	0.5	**Rec**	(1.0, 2.2)	0.04665 ^∗^	**0.84330**	0.63678	0.76013	0.50830	0.63486	
				**Mult**	(1.8, 3.24)	0.44423 ^∗^	0.62624 ^∗^	**0.82447**	0.74065	0.81753	0.82465	
				**Unr**	(0.65, 1.54)	0.24177	0.87274	0.30563	**0.84850**	0.15914	0.30947	
				**Dom**	(2.6, 2.6)	**0.96437**	0.08869	0.56774	0.92870	0.79380		
		25	0.5	**Rec**	(1.0, 2.2)	0.05605	**0.95407**	0.76586	0.91508	0.61764		
				**Mult**	(1.8, 3.24)	0.49237	0.71043	**0.92158**	0.86355	0.91256		
50	100			**Unr**	(0.65, 1.54)	0.55513	0.98255	0.38476	**0.98123**	0.17861		
				**Dom**	(2.6, 2.6)	**0.93871**	0.07985	0.51548	0.88896	0.74713		
		0	0.5	**Rec**	(1.0, 2.2)	0.05134	**0.92769**	0.71809	0.87193	0.55984		
				**Mult**	(1.8, 3.24)	0.45734	0.66902	**0.88925**	0.82026	0.87906		
				**Unr**	(0.65, 1.54)	0.47192	0.96392	0.34894	**0.96087**	0.16023		
				**Dom**	(2.2, 2.2)	**0.89874**	0.05076	0.76992	0.83080	0.84520		0.53247
		25	0.2	**Rec**	(1.0, 3.6)	0.03613	**0.92147**	0.48451	0.87037	0.30487		0.31694
				**Mult**	(1.9, 3.61)	0.69925	0.34702	**0.85778**	0.77836	0.85218		0.62966
0	100			**Unr**	(0.5, 2.0)	0.65567	0.84785	0.10276	**0.94265**	0.03027		0.08794
				**Dom**	(2.2, 2.2)	**0.84384**	0.04086 ^∗^	0.70946	0.75109	0.78697	0.70494	
		0	0.2	**Rec**	(1.0, 3.6)	0.03782 ^∗^	**0.86147**	0.43153	0.79210	0.26755	0.42869	
				**Mult**	(1.9, 3.61)	0.64051 ^∗^	0.32123 ^∗^	**0.80066**	0.70908	0.79017	0.79740	
				**Unr**	(0.5, 2.0)	0.54538	0.75786	0.10187	**0.87870**	0.02940	0.09909	
				**Dom**	(2.2, 2.2)	**0.94780**	0.08895	0.82800	0.90217	0.90049		
		25	0.2	**Rec**	(1.0, 3.6)	0.05440	**0.97394**	0.53918	0.95030	0.31670		
				**Mult**	(1.9, 3.61)	0.72728	0.34717	**0.90222**	0.83592	0.89492		
50	100			**Unr**	(0.5, 2.0)	0.83495	0.95478	0.10560	**0.98840**	0.02343		
				**Dom**	(2.2, 2.2)	**0.91980**	0.07593	0.78331	0.85809	0.86423		
		0	0.2	**Rec**	(1.0, 3.6)	0.04721	**0.95163**	0.49510	0.91409	0.28013		
				**Mult**	(1.9, 3.61)	0.67942	0.32597	**0.86532**	0.78885	0.85618		
				**Unr**	(0.5, 2.0)	0.77111	0.92279	0.10152	**0.97278**	0.02310		
				**Dom**	(2.2, 2.2)	**0.57438**	0.02821	0.53632	0.43550	0.56002		0.33291
		25	0.05	**Rec**	(1.0, 3.6)	0.04380	**0.29455**	0.06729	0.21332	0.04873		0.06352
				**Mult**	(1.9, 3.61)	0.38400	0.10189	**0.42547**	0.34874	0.41536		0.26377
0	100			**Unr**	(0.5, 2.0)	0.22082	0.27767	0.18594	**0.32682**	0.12097		0.14033
				**Dom**	(2.2, 2.2)	**0.50622**	0.03413	0.47104	0.39360	0.49103	0.46796	
		0	0.05	**Rec**	(1.0, 3.6)	0.04448	**0.27724**	0.06733	0.20273	0.04704	0.06674	
				**Mult**	(1.9, 3.61)	0.33877	0.10807	**0.36803**	0.31909	0.36089	0.36674	
				**Unr**	(0.5, 2.0)	0.17709	0.25044	0.15031	**0.26490**	0.06971	0.17677	
				**Dom**	(2.2, 2.2)	**0.63920**	0.01453	0.58425	0.47921	0.62697		
		25	0.05	**Rec**	(1.0, 3.6)	0.04465	**0.28842**	0.06893	0.23235	0.04345		
				**Mult**	(1.9, 3.61)	0.42156	0.08232	**0.46540**	0.36949	0.46364		
50	100			**Unr**	(0.5, 2.0)	0.30208	0.31549	0.20745	**0.42153**	0.15479		
				**Dom**	(2.2, 2.2)	**0.58412**	0.01814	0.54315	0.42708	0.57007		
		0	0.05	**Rec**	(1.0, 3.6)	0.04222	**0.29220**	0.06943	0.22264	0.04549		
				**Mult**	(1.9, 3.61)	0.38029	0.08648	**0.42651**	0.33479	0.41893		
				**Unr**	(0.5, 2.0)	0.27336	0.30692	0.18868	**0.37802**	0.12098		

∗ indicates entry that is slightly lower than the corresponding entry of Table three, Troendle et al. [19].

**Table 3:** **Power performance of the proposed tests at 0.05 nominal significance level using the parameter in Table three, Troendle et al. **[19]**and *n = 500***

**Sample size**			**p**	**Disease model**		**Model**					**TDT**	***z***_***com***_
**s**	**n**	**m**		**Model**	**(*ψ***_**1**_**,*ψ***_**2**_**)**	**Dom**	**Rec**	**Mult**	**Unr**	**Add**		
				**Dom**	(1.5, 1.5)	**0.91984**	0.05494	0.59684	0.86250	0.67998		0.38389
		125	0.5	**Rec**	(1.0, 1.45)	0.04830	**0.91488**	0.68434	0.85930	0.61349		0.46383
				**Mult**	(1.3, 1.69)	0.54475	0.63588	**0.88638**	0.81607	0.88395		0.67525
0	500			**Unr**	(0.82, 1.22)	0.40678	0.94369	0.28652	**0.95067**	0.19831		0.19157
				**Dom**	(1.5, 1.5)	**0.86469**	**0.05501**^**∗**^	0.53494	0.78362	0.61161	0.53133	
		0	0.5	**Rec**	(1.0, 1.45)	**0.04868**^**∗**^	**0.86048**	0.62189	0.78284	0.54995	0.61559	
				**Mult**	(1.3, 1.69)	**0.50217**^**∗**^	**0.58591**^**∗**^	**0.83504**	0.74791	0.82951	0.83316	
				**Unr**	(0.82, 1.22)	0.30751	0.88989	0.25717	**0.89710**	0.18219	0.25521	
				**Dom**	(1.5, 1.5)	**0.93417**	0.05975	0.61089	0.88359	0.69680		
		125	0.5	**Rec**	(1.0, 1.45)	0.04970	**0.93022**	0.70132	0.87754	0.62959		
				**Mult**	(1.3, 1.69)	0.55266	0.64473	**0.89571**	0.83059	0.89378		
50	500			**Unr**	(0.82, 1.22)	0.46190	0.95843	0.29617	**0.96292**	0.20571		
				**Dom**	(1.5, 1.5)	**0.88543**	0.05450	0.55038	0.81938	0.63176		
		0	0.5	**Rec**	(1.0, 1.45)	0.04813	**0.88221**	0.63748	0.81229	0.56987		
				**Mult**	(1.3, 1.69)	0.50941	0.59514	**0.85057**	0.77031	0.84857		
				**Unr**	(0.82, 1.22)	0.35793	0.91611	0.26407	**0.92113**	0.18285		
				**Dom**	(1.45, 1.45)	**0.91663**	0.05581	0.82275	0.85564	0.85475		0.59268
		125	0.2	**Rec**	(1.0, 2.0)	0.04455	**0.94790**	0.44326	0.90851	0.34805		0.28523
				**Mult**	(1.35, 1.82)	0.74330	0.29700	**0.86030**	0.77766	0.85735		0.63475
0	500			**Unr**	(0.73, 1.37)	0.75736	0.83819	0.16262	**0.95415**	0.09838		0.11611
				**Dom**	(1.45, 1.45)	**0.86430**	**0.05206**^**∗**^	0.76184	0.78832	0.79620	0.76041	
		0	0.2	**Rec**	(1.0, 2.0)	**0.04254**^**∗**^	**0.90108**	0.39325	0.84045	0.30808	0.38860	
				**Mult**	(1.35, 1.82)	**0.67838**^**∗**^	**0.27300**^**∗**^	**0.79869**	0.70763	0.79626	0.79935	
				**Unr**	(0.73, 1.37)	0.65414	0.73828	0.14995	**0.90150**	0.09179	0.14809	
				**Dom**	(1.45, 1.45)	**0.92776**	0.05929	0.83556	0.87192	0.86570		
		125	0.2	**Rec**	(1.0, 2.0)	0.04580	**0.95984**	0.45325	0.92594	0.35732		
				**Mult**	(1.35, 1.82)	0.75081	0.29775	**0.86878**	0.79374	0.86823		
50	500			**Unr**	(0.73, 1.37)	0.79247	0.87274	0.17039	**0.96622**	0.09941		
				**Dom**	(1.45, 1.45)	**0.88237**	0.05344	0.78154	0.81038	0.81656		
		0	0.2	**Rec**	(1.0, 2.0)	0.04352	**0.92016**	0.40450	0.87014	0.31454		
				**Mult**	(1.35, 1.82)	0.69361	0.27258	**0.81773**	0.72982	0.81359		
				**Unr**	(0.73, 1.37)	0.69897	0.78788	0.15242	**0.92672**	0.09045		
				**Dom**	(1.45, 1.45)	**0.54540**	0.01899	0.51668	0.41742	0.53330		0.32711
		125	0.05	**Rec**	(1.0, 2.0)	0.04787	**0.19369**	0.06095	0.15167	0.04953		0.05595
				**Mult**	(1.35, 1.82)	0.37363	0.06127	**0.39755**	0.30298	0.39613		0.25117
0	500			**Unr**	(0.73, 1.37)	0.32954	0.14447	0.25594	**0.32487**	0.23685		0.16860
				**Dom**	(1.45, 1.45)	**0.47895**	0.02234	0.45528	0.35409	0.46737	0.45051	
		0	0.05	**Rec**	(1.0, 2.0)	0.04759	**0.17392**	0.06026	0.13605	0.04958	0.05914	
				**Mult**	(1.35, 1.82)	0.32514	0.06317	**0.34478**	0.26105	0.34585	0.34717	
				**Unr**	(0.73, 1.37)	0.28430	0.12649	0.22702	**0.27964**	0.20895	0.22606	
				**Dom**	(1.45, 1.45)	**0.55918**	0.01811	0.53173	0.43260	0.54426		
		125	0.05	**Rec**	(1.0, 2.0)	0.04848	**0.20376**	0.06147	0.15963	0.04985		
				**Mult**	(1.35, 1.82)	0.38246	0.06150	**0.41190**	0.31308	0.40672		
50	500			**Unr**	(0.73, 1.37)	0.34311	0.14948	0.27061	**0.34266**	0.22740		
				**Dom**	(1.45, 1.45)	**0.49431**	0.02036	0.46935	0.36889	0.48220		
		0	0.05	**Rec**	(1.0, 2.0)	0.04777	**0.17937**	0.06071	0.14178	0.05204		
				**Mult**	(1.35, 1.82)	0.33955	0.06127	**0.36054**	0.26850	0.35641		
				**Unr**	(0.73, 1.37)	0.29572	0.13457	0.23367	**0.29240**	0.20284		

∗ indicates entry that is slightly lower than the corresponding entry of Table three, Troendle et al. [19].

In Table 3, the empirical powers of *Mult* model are close to those of *Add* model. In the first *Mult* model, the parameters are *ψ*_**1**_**=1.3 and ***ψ*_**2**_**=1.69; in the second***Mult* model, the parameters are *ψ*_**1**_**=1.35 and***ψ*_**2**_**=1.82; for both cases,***ψ*_**2**_**=2***ψ*_**1**_−1 is roughly true, and so it leads to similar results for the two models.

### Example: cleft palate data of *TGFA* gene of Irish study

We applied the proposed methods to examine the association between oral clefts and the *TGFA* gene in the Irish study [1]. We focused on cleft palate only. The data were ascertained through the presence of a cleft palate in the child, and so the ascertainment procedure satisfies our model assumption. In the dataset, there are 31 SNPs in 12 candidate genes. One SNP, rs2166975, is located in the region of the *TGFA* gene. In Carter et al. [1], SNP rs2166975 was found to be associated with cleft palate by transmission disequilibrium test based on triad families (*p*-value = 0.041). For the SNP rs2166975, there are 296 triad counts, 62 parent-child dyads, and 15 affected monads in our analysis.

The results of the proposed likelihood ratio tests of SNP rs2166975 are presented in Table 4. Using the 296 full triads, the tests of *Dom* and *Add* show significant signals of association between SNP rs2166975 and cleft palate (*p*-value = 0.047 and 0.05, respectively). By using 296 full triads, 62 parent-child dyads, and 15 affected monads, the test of *Mult* also shows significant signal of association (*p*-value = 0.051) and the test of *Add* suggestively shows it (*p*-value = 0.055). Using the 296 full triads, the test of *Mult* suggestively shows the signal (*p*-value = 0.058). Table 4 provides results of all five tests (*Unr, Rec, Dom*, *Mult*, and *Add*) and parameter MLEs for each model.

**Table 4:** **Results of the proposed likelihood ratio tests of SNP rs2166975 and parameter estimates in the region of gene*TGFA* for cleft palate only data in Carter et al. [**[1]**]**

**Model**	**Data used**	**MLEs**		**Test results**	
**Unr**		$\hat{p}$	($\tilde{p}$, $\tilde{\psi_{1}}$, ${\tilde{\psi }}_{2}$)	**LRT**	**p-value**
	**FT + PD + AM**	0.250	(0.226, 1.260, 1.660)	3.821	0.148
	**FT + PD**	0.246	(0.226, 1.279, 1.413)	2.994	0.224
	**FT**	0.245	(0.221, 1.365,1.508)	4.077	0.130
**Rec**		$\hat{p}$	($\tilde{p},{\tilde{\psi }}_{2}$)	**LRT**	**p-value**
	**FT + PD + AM**	0.250	(0.243, 1.295)	1.245	0.265
	**FT + PD**	0.246	(0.244, 1.087)	0.116	0.733
	**FT**	0.245	(0.243, 1.095)	0.113	0.737
**Dom**		$\hat{p}$	($\tilde{p},{\tilde{\psi }}_{1})$	**LRT**	**p-value**
	**FT + PD + AM**	0.250	(0.233, 1.247)	2.407	0.121
	**FT + PD**	0.246	(0.228, 1.275)	2.829	0.093
	**FT**	0.245	(0.224, 1.362)	3.938	0.047
**Mult**		$\hat{p}$	($\tilde{p},{\tilde{\psi }}_{1})$	**LRT**	**p-value**
	**FT + PD + AM**	0.250	(0.226, 1.275)	3.792	0.051
	**FT + PD**	0.246	(0.226, 1.232)	2.711	0.100
	**FT**	0.245	(0.221, 1.292)	3.585	0.058
**Add**		$\hat{p}$	($\tilde{p},{\tilde{\psi }}_{1})$	**LRT**	**p-value**
	**FT + PD + AM**	0.250	(0.226, 1.281)	3.688	0.055
	**FT + PD**	0.246	(0.225, 1.256)	2.847	0.092
	**FT**	0.245	(0.220, 1.332)	3.830	0.050

The Sub-sample sizes are *n* = 296, *m* = 62, *s* = 15, (*n*_**1**_, *n*_**2**_, *n*_**3**_, *n*_**4**_, *n*_**5**_, *n*_**6**_, *n*_**7**_, *n*_**8**_, *n*_**9**_, *n*_**10**_) = (0, 6, 5, 19, 13, 30, 6, 67, 54, 96), (*m*_**1**_, *m*_**2**_, *m*_**3**_, *m*_**4**_, *m*_**5**_, *m*_**6**_, *m*_**7**_) = (0, 3, 4, 11,11, 8, 25), and (*s*0, *s*1, *s*2) = (7, 3, 5). **Abbreviations**: *FT* = Full triads, *PD* = Parent-child dyads, and *AM* = Affected monads.

SNP rs2166975 in *TGFA* gene is the top one using both TDT and the LRTs of the proposed models. The results of the proposed models are consistent with that of TDT based on triad families. The reported association between the *TGFA* gene and cleft palate is confirmed by the proposed *Dom*, *Add*, and *Mult* models. However, the *p*-values of the LRTs of the proposed *Rec* and *Unr* models are not significant at a cutoff of 0.05. In summary, the association between *TGFA* gene and cleft palate is only confirmed by 3 out of 5 proposed models. This is expected since it is unlikely that all models can give significant results.

### Computational evaluation based on the cleft palate data of *TGFA* gene of the Irish study

The dataset of Carter et al. [1] is not from a genome-wide association study (GWAS). To get the results for the 31 SNPs of the cleft palate data of the Irish study, it takes about 4 minutes on our PC computers. Based on our evaluation, it takes about one hour to analyze 450 SNPs by the proposed models if the sample size of the data is similar to that of the dataset of Carter et al. [1]. In 24 hours, the proposed models can analyze about 10,000 SNPs. Therefore, the proposed models are slower than TDT. This is because we need to estimate the parameters in our models by doing maximum likelihood estimations. The proposed models are not suggested for GWAS analysis which has millions of SNPs. For GWAS which has millions of SNPs, one may want to run TDT first. The proposed models can be used as a follow-up to confirm the association for the SNPs in the candidate gene regions.

## Discussion

In this paper, we construct the likelihood directly by using the results in Tables 5 and 6, and we argue that imputation is not necessary to deal with the missingness of parent data. Furthermore, standard statistical methods such as Newton-Raphson can be used to estimate the parameters. Note that this facilitates data analysis and interpretation a lot and computationally it is much easier.

**Table 5:** Conditional probabilities of parental mating type and triad genotypes given the sampling scheme of using the affected child as a proband

**Parental**	**Affected child**	**P(MT, C | D)**	**P(MT | D)**	**# Obs**
**mating type**	**genotype C**			
1. AA × AA	AA	*p*^**4**^*ψ*_**2**_/*R*	*p*^**4**^*ψ*_**2**_/*R*	*n*_**1**_
2. AA × Aa	AA	4*p*^**3**^*qψ*_**2**_/(2*R*)	$4p^{3}q\frac{\psi_{1}+\psi_{2}}{2R}$	*n*_**2**_
	Aa	4*p*^**3**^*qψ*_**1**_/(2*R*)		*n*_**3**_
3. AA × aa	Aa	2*p*^**2**^*q*^**2**^*ψ*_**1**_/*R*	2*p*^**2**^*q*^**2**^*ψ*_**1**_/*R*	*n*_**4**_
4. Aa × Aa	AA	4*p*^**2**^*q*^**2**^*ψ*_**2**_/(4*R*)	$4p^{2}q^{2}\frac{\psi_{2}+2\psi_{1}+1}{4R}$	*n*_**5**_
	Aa	4*p*^**2**^*q*^**2**^(2*ψ*_**1**_)/(4*R*)		*n*_**6**_
	aa	4*p*^**2**^*q*^**2**^/(4*R*)		*n*_**7**_
5. Aa × aa	Aa	4*pq*^**3**^*ψ*_**1**_/(2*R*)	$4pq^{3}\frac{\psi_{1}+1}{2R}$	*n*_**8**_
	aa	4*pq*^**3**^/(2*R*)		*n*_**9**_
6. aa × aa	aa	*q*^**4**^/*R*	*q*^**4**^/*R*	*n*_**10**_
**Total**		1	1	*n*

**Abbreviation**: *MT* = Mating type, *Obs* = Observation. *R* =*p*^**2**^*ψ*_**2**_ + 2*pqψ*_**1**_ + *q*^**2**^.

**Table 6:** Conditional probabilities of parent-child dyad genotypes given the sampling scheme of using the affected child as a proband

**Genotype**		**P(G, C ∣ D), G = M or F,**	**P(G, C ∣ D)**,	**# Obs**
**Parent*G***	**Case*C***	**complex version**	**simplified version**	
AA	AA	*p*^**4**^*ψ*_**2**_/*R* + 2*p*^**3**^*qψ*_**2**_/(2*R*)	*p*^**3**^*ψ*_**2**_/*R*	*m*_**1**_
	Aa	2*p*^**3**^*qψ*_**1**_/(2*R*) + *p*^**2**^*q*^**2**^*ψ*_**1**_/*R*	*p*^**2**^*qψ*_**1**_/*R*	*m*_**2**_
	AA	2*p*^**3**^*qψ*_**2**_/(2*R*) + 4*p*^**2**^*q*^**2**^*ψ*_**2**_/(4*R*)	*p*^**2**^*qψ*_**2**_/*R*	*m*_**3**_
Aa	Aa	2*p*^**3**^*qψ*_**1**_/(2*R*) + 4*p*^**2**^*q*^**2**^(2*ψ*_**1**_)/(4*R*) + 2*pq*^**3**^*ψ*_**1**_/(2*R*)	*pqψ*_**1**_/*R*	*m*_**4**_
	aa	4*p*^**2**^*q*^**2**^/(4*R*) + 2*pq*^**3**^/(2*R*)	*pq*^**2**^/*R*	*m*_**5**_
aa	Aa	*p*^**2**^*q*^**2**^*ψ*_**1**_/*R* + 2*pq*^**3**^*ψ*_**1**_/(2*R*)	*pq*^**2**^*ψ*_**1**_/*R*	*m*_**6**_
	aa	2*pq*^**3**^/(2*R*) + *q*^**4**^/*R*	*q*^**3**^/*R*	*m*_**7**_
**Total**		1	1	*m*

**Abbreviation**: *Obs* = Observation.

Although the proposed models are built to analyze combinations of triad families, dyad data, and affected monads, it is possible to extend them to analyze other types of family data, e.g., family data with multiple offspring, sibship data, and general pedigrees. To combine different types of family data in the analysis, one needs to take the ascertained procedure into account and build the likelihood. For general pedigree data, the imputation procedure and methods proposed by other researchers such as Epstein et al. [11], McPeek [20], and Weinberg [4] can be very useful. By a combined analysis of all family data, it takes advantage of the robustness of family studies to avoid high false positive rates and it improves power since more data are used in the analysis. For data with a relatively simple structure such as combinations of full triads, parent-child dyads, and affected singleton monads, however, the proposed methods in this article are straightforward and easy to implement for genetic community without imputation.

The impact of important issues on the proposed methods such as population stratification and heterogeneity are not investigated in the current study. This is because the data structure of our oral clefts study is relatively homogeneous since our project focused on an Irish population and was carefully designed to make sure the data are homogeneous. Therefore, we may calculate the likelihood directly to avoid computational complexity. In the presence of population stratification and heterogeneity, sophisticated models can be built to analyze the data [5]**,**[21]**-**[23]. For instance, if the data are from two sub-populations with different allele frequencies, the conditional probabilities of mating type *P*(*MT*=*i*∣*D*) can be modified to accommodate the population stratification. Then, the corresponding likelihood functions can be calculated to test for association between disease trait and genetic marker. In addition, we only use one di-allelic genetic marker in the analysis and we do not use environment factors. It is important to develop a method to add more genetic variants and environment factors to the models. Then, we may be able to investigate the impact of gene-gene and gene-environment interactions. These are interesting problems to investigate in the future studies.

## Conclusion

In this paper, we develop likelihood-based statistical models and likelihood ratio tests to test association between complex diseases and genetic markers by using combinations of full triads, parent-child dyads, and affected singleton monads for a unified analysis. For the data we discuss, a likelihood can be calculated directly to facilitate the data analysis without imputation [11]. This makes it easy to implement the models and to explain the results. By simulation studies, we show that the proposed models and tests are very robust in terms of type I error evaluations, and are powerful by empirical power evaluations. The methods are applied to analyze cleft palate data of the *TGFA* gene of an Irish study to show the association found previously [1].

## Methods

### Likelihoods

Consider a design which includes three types of data: (1) *n* triad families each consists of an affected child and two parents; (2) *m* parent-child dyads with an affected child and a parent who can be either father or mother; (3) *s* affected singleton monads. The triads, parent-child dyads, and the affected singleton monads are ascertained through the affected cases. Suppose we have a di-allelic candidate gene locus which has two alleles *A* and *a* with allele frequencies *p* and *q*, respectively. Let *D* denote that an individual is affected with the disease. Given the disease status, let us define the disease penetrance as *f*_**2**_=*P*(*D*|*AA*),*f*_**1**_=*P*(*D*|*Aa*) and *f*_**0**_=*P*(*D*|*aa*). Such as Schaid and Sommer [12], define the relative risks as *ψ*_**2**_=*f*_**2**_/*f*_**0**_ and *ψ*_**1**_=*f*_**1**_/*f*_**0**_.

For triads, let us denote the genotypes at the candidate gene locus as *F*,*M*, and *C*, where *F* is the genotype of the father, *M* is the genotype of the mother, and *C* is the genotype of the affected child. In total, there are 6 mating types [12]**,**[24]. Let us denote MT = {mating type}. Assume that Hardy-Weinberg equilibrium (HWE) is valid. We also assume random mating in the parental generation. Given the sampling scheme of using the affected child as a proband, the conditional probabilities of mating type *P*(MT=*i*|*D*) and the conditional probabilities of mating type and child genotype *P*(MT=*i*,*C*|*D*) can be derived as

$$\begin{array}{ll} P(\text{MT}=i,C|D) & =\frac{P(\text{MT}=i,C,D)}{P(D)}\\ & =\frac{P(\text{MT}\;=\;i)P(C|\text{MT}\;=\;i)P(D|C,\;\text{MT}=i)}{P(D)}\\ & =\frac{P(\text{MT}=i)P(C|\text{MT}=i)P(D|C)}{P(D)},\\ P(\text{MT}=i|D) & =P(\text{MT}=i)\left[P(\text{AA}|\text{MT}=i\;)\psi_{2}\right)\\ & \;\left(+P(\text{Aa}|\text{MT}\;=\;i)\psi_{1}\;+\;P(\text{aa}|\text{MT}=i)\right]/R, \end{array}$$

where *R*=*p*^**2**^*ψ*_**2**_+2*pqψ*_**1**_+*q*^**2**^, *C*=*AA*,*Aa*,*aa*, and *i*=1,2,⋯,6. There are 10 combinations (MT=*i*,*C*). The results are presented in Table 5, which are the same as those of Table one in Nagelkerke et al. [13]. Using the notation given in Table 5, we have the following log-likelihood

(1) $$\begin{array}{lll} \log L_{\text{Triads}} & =\left[4n_{1}+3n_{2}+3n_{3}+2n_{4}+2n_{5}+2n_{6}\right)\; & \;\\ & \;\left(+2n_{7}+n_{8}+n_{9}\right]\log p\; & \;\\ & \;+\left[n_{2}+n_{3}+2n_{4}+2n_{5}+2n_{6}+2n_{7}\right)\; & \;\\ & \;\left(+3n_{8}+3n_{9}+4n_{10}\right]\log q\; & \;\\ & \;+\left[n_{3}+n_{4}+n_{6}+n_{8}\right]\log\psi_{1}\; & \;\\ & \;+\left[n_{1}+n_{2}+n_{5}\right]\log\psi_{2}-n\log R,\; \end{array}$$

where *n*_*i*_ are sub-sample sizes of the ten entries in Table 5.

For parent-child dyads, denote the genotypes at the candidate gene locus as *G* and *C*, where *G*=*M* or *G*=*F* is the genotype of the parent and *C* is the genotype of the affected child. Given the sampling scheme of using the affected child as a proband, the conditional probabilities of parent-child pair genotypes *P*(*M*,*C*|*D*) can be derived. For instance, we may calculate

(2) $$\begin{array}{lll} \;P(M\;=\;\text{Aa},C\;=\;\text{AA}|D) & =P(M=\text{Aa},F=\text{AA},C=\text{AA}|D)\; & \;\\ & \;+P(M\;=\;\text{Aa},F=\text{Aa},C=\text{AA}|D)\; & \;\\ & =P(\text{MT}\;=\;\text{Aa}\times \text{AA},C=\text{AA}|D)/2\; & \;\\ & \;+P(\text{MT}\;=\;\text{Aa}\times \text{Aa},C=\text{AA}|D)\; & \;\\ & =2p^{3}q\psi_{2}/(2R)+4p^{2}q^{2}\psi_{2}/(4R)\; & \;\\ & =p^{2}q\psi_{2}/\mathrm{R.}\; \end{array}$$

Table 6 presents the possible conditional probabilities of 7 parent-child dyads. Then, we have the log-likelihood

(3) $$\begin{array}{lll} \;\log L_{\text{Parent}-\text{Child}-\text{Dyads}} & =\left[3m_{1}+2m_{2}+2m_{3}+m_{4}\right)\; & \;\\ & \;\left(+m_{5}+m_{6}\right]\log p\; & \;\\ & \;+\left[m_{2}+m_{3}+m_{4}+2m_{5}\right)\; & \;\\ & \;\left(+2m_{6}+3m_{7}\right]\log q\; & \;\\ & \;+\left[m_{2}+m_{4}+m_{6}\right]\log\psi_{1}\; & \;\\ & \;+\left[m_{1}+m_{3}\right]\log\psi_{2}-m\log\mathrm{R.}\; \end{array}$$

Our derivation above in (2) is different from that of Schaid and Sommer [12], p1119, right column, lines 11–12 from bottom. Schaid and Sommer [12] considered that “a case has genotype *AA*, one of its parents has genotype *Aa*, and the genotype of the other parent is missing” which does not specify which parents, father or mother, having *Aa* or missing genotypes. However, the present paper takes an ordered example of mother having *Aa* genotype and father having missing genotype, which is different from the unordered case in Schaid and Sommer [12]. To make it clear, Schaid and Sommer [12] calculated

(4) $$\begin{array}{lll} P(M & =\text{Aa}\text{or}F=\text{Aa},C=\text{AA}|D)\; & \;\\ & =P(M=\text{Aa},F=\text{AA},C=\text{AA}|D)\; & \;\\ & \;+P(M=\text{AA},F=\text{Aa},C=\text{AA}|D)\; & \;\\ & \;+P(M=\text{Aa},F=\text{Aa},C=\text{AA}|D)\; & \;\\ & =P(\text{MT}=\text{Aa}\times \text{AA},C=\text{AA}|D)\; & \;\\ & \;+P(\text{MT}=\text{Aa}\times \text{Aa},C=\text{AA}|D)\; & \;\\ & =2p^{3}q\psi_{2}/R+4p^{2}q^{2}\psi_{2}/(4R)\; & \;\\ & =p^{2}q\psi_{2}/R+p^{3}q\psi_{2}/\mathrm{R.}\; \end{array}$$

In practice it is easy to make mistakes by applying the unordered result like (4) in Schaid and Sommer [12] directly, since in data it is usually an ordered case.

For the *s* affected singleton monads, assume *s*_**2**_ of them have genotype *AA*, *s*_**1**_ of them have genotype *Aa*, and *s*_**0**_ of them have genotype *aa* (*s*_**2**_+*s*_**1**_+*s*_**0**_=*s*). Then, *P*(*AA*|*D*)=*p*^**2**^*ψ*_**2**_/*R*, *P*(*Aa*|*D*)=2*pqψ*_**1**_/*R*, and *P*(*aa*|*D*)=*q*^**2**^/*R*. Let us denote the likelihood of the affected singleton monads as *L*_*affected*−*Monads*_. Then, we have the following log-likelihood

(5) $$\begin{array}{lll} \;\log L_{\text{Affected}-\text{Monads}} & =\;[\;2s_{2}+s_{1}]\log p\;+\;[\;s_{1}+2s_{0}]\log q\; & \;\\ & \;+s_{1}\log\psi_{1}+s_{2}\log\psi_{2}-s\log\mathrm{R.}\; \end{array}$$

Based on the three log-likelihoods (1), (3), and (5), we may calculate the log-likelihood of full data as follows

$$\begin{array}{lll} \log L(\psi_{1},\psi_{2},p) & =\log L_{\text{Triads}}+\log L_{\text{Parent}-\text{Child}-\text{Dyads}}\; & \;\\ & \;+\log L_{\text{Affected}-\text{Monads}}.\; & \; \end{array}$$

### Likelihood ratio tests of genetic association

Under the null hypothesis of no association between the disease and the marker locus, we have *H*_**0**_:*ψ*_**1**_=*ψ*_**2**_=1. There is only one parameter *p* to estimate under the null hypothesis *H*_**0**_ and the log-likelihood is equal to $\log L(1,1,\hat{p})$, and $\hat{p}$ is the maximum likelihood estimate (MLE) of *p*.

Without any restrictive condition on the parameters, one gets an unrestricted alternative hypothesis *H*_*Unr*_:*ψ*_**1**_≥0,*ψ*_**2**_≥0. Let ${\tilde{\psi }}_{1},{\tilde{\psi }}_{2}$ and $\tilde{p}$ be the MLE of *ψ*_**1**_,*ψ*_**2**_ and *p* under *H*_*Unr*_. The likelihood ratio test statistic of association is

$$\begin{array}{lll} \text{Unr} & =2\log L({\tilde{\psi }}_{1},{\tilde{\psi }}_{2},\tilde{p})-2\log L(1,1,\hat{p}).\; & \; \end{array}$$

*Unr* is approximately chi-square distributed with 2 degrees of freedom (DF) by large sample theory, when the sample size is sufficiently large.

Under a dominant model, one imposes a restriction of an alternative hypothesis *H*_*Dom*_:*ψ*_**1**_=*ψ*_**2**_. Let ${\tilde{\psi }}_{1}$ and $\tilde{p}$ be the MLE of *ψ*_**1**_ and *p* under *H*_*Dom*_, respectively. The LRT of association is

$$\begin{array}{lll} \text{Dom} & =2\log L({\tilde{\psi }}_{1},{\tilde{\psi }}_{1},\tilde{p})-2\log L(1,1,\hat{p}).\; & \; \end{array}$$

If a recessive disease model is desired, one has an alternative hypothesis *H*_*Rec*_:*ψ*_**1**_=1,*ψ*_**2**_≥0. Let ${\tilde{\psi }}_{2}$ and $\tilde{p}$ be the MLE of *ψ*_**2**_ and *p* under *H*_*Rec*_, respectively. The LRT of association is

$$\begin{array}{lll} \text{Rec} & =2\log L(1,{\tilde{\psi }}_{2},\tilde{p})-2\log L(1,1,\hat{p}).\; & \; \end{array}$$

Under a multiplicative model, an alternative hypothesis is $H_{\text{Mult}}:\psi_{2}=\psi_{1}^{2}$. Let ${\tilde{\psi }}_{1}$ and $\tilde{p}$ be the MLE of *ψ*_**1**_ and *p* under *H*_*Mult*_, respectively. The LRT of association is

$$\begin{array}{lll} \text{Mult} & =2\log L({\tilde{\psi }}_{1},{\tilde{\psi }}_{1}^{2},\tilde{p})-2\log L(1,1,\hat{p}).\; & \; \end{array}$$

If an additive model is used, one has an alternative hypothesis *H*_*Add*_:*ψ*_**2**_=2*ψ*_**1**_−1. Let ${\tilde{\psi }}_{1}$ and $\tilde{p}$ be the MLE of *ψ*_**1**_ and *p* under *H*_*Add*_, respectively. The LRT of association is

$$\begin{array}{lll} \text{Add} & =2\log L({\tilde{\psi }}_{1},2{\tilde{\psi }}_{1}-1,\tilde{p})-2\log L(1,1,\hat{p}).\; & \; \end{array}$$

*Dom*, *Rec*, *Mult*, and *Add* are approximately chi-square distributed with 1 DF by large sample theory, when the sample size is sufficiently large. In Appendix-A of Additional file 1, we provide procedures and formulae to perform MLE and LRT calculations by Newton-Raphson methods.

### Transmission disequilibrium tests

Using the notations in Table 5, it can be shown that the transmission disequilibrium test (TDT) based on triads is *TDT*=(*b*−*c*)^**2**^/(*b*+*c*), where *b*=*n*_**2**_+2*n*_**5**_+*n*_**6**_+*n*_**8**_ and *c*=*n*_**3**_+*n*_**6**_+2*n*_**7**_+*n*_**9**_[3]. Combining both triads and parent-child dyads and using the notations in Table 5 and Table 6, we may define a score test $z_{\text{com}}=(W\;-\;A_{\text{com}})/\sqrt{V_{\text{com}}}$, where *W*=*b*+*b*_**1**_,*A*_*com*_=(*b*+*c*)/2+(*b*_**1**_+*c*_**1**_)/2,*V*_*com*_=(*b*+*c*)/4+(*b*_**1**_+*c*_**1**_)/4,*b*_**1**_=*m*_**2**_+*m*_**5**_, and *c*_**1**_=*m*_**3**_+*m*_**6**_[10]. By large sample theory, the *TDT* is approximately chi-square distributed with 1 DF and *z*_*com*_ is approximately normally distributed when the sample size is sufficiently large.

### Simulations

In our simulation, we use the same notations as those in the section of **Models**. For instance, *p* is the allele frequency of allele *A*,*n* is the number of triad families, *m* is the number of dyad families, and *s* is the number of monads. Hence, *n*,*m*, and *s* are sample sizes for triads, dyads, and monads, respectively.

For power calculations, the data are simulated under disease models using the multinomial distribution. For instance, let us look at the upper left corner cell 0.84541 of empirical power in Table 2. The cell corresponds to a sample size *n*=100 of triad families, a sample size *m*=25 of dyads and no monads *s*=0, a given allele frequency *p*=0.5, and parameters *ψ*_**1**_=*ψ*_**2**_=2.6. By using the 10 probabilities of Table 5 in column 3 based on given allele frequency *p*=0.5 and parameters *ψ*_**1**_=*ψ*_**2**_=2.6, we generate the triad counts $n_{i},i=1,\cdots \;,10,\underset{i}{\sum }n_{i}=100,$ under the multinomial distribution. The same strategy applies to generate dyad data $m_{1},\cdots \;,m_{7},\underset{i}{\sum }m_{i}=25,$ by using the 7 probabilities of Table 6. These counts are then combined to estimate the parameters and calculate the likelihood test *Unr* using unrestricted model. The process is repeated 100,000 times. The number 0.84541 is the the proportion of the *Unr* test values calculated for the 100,000 samples, that exceed the 95-th percentiles of the $\chi_{2}^{2}$-distribution. For type I error calculation, the parameters *ψ*_**1**_ and *ψ*_**2**_ are taken to be 1 under the null hypothesis of no association using the multinomial distribution.

## Competing interests

The authors declare that they have no competing interests.

## Authors’ contributions

RF, AL, JFT, and JLM contributed to the conception and design of the study, analysis and interpretation of data, and AL and ZL wrote the programs, and performed the simulation analysis. All authors have been involved in writing the manuscript and approved this final version. All authors read and approved the final manuscript.

## Supplementary Material

Additional file 1 Supplementary material. Click here for file
