# Supplementary material for: Association analysis of complex diseases using triads, parent-child dyads and singleton monads
Source: BMC Genet. 2013 Sep 4;14:78. doi: 10.1186/1471-2156-14-78 (PMC3844511; doi:10.1186/1471-2156-14-78)
Supplement: Additional file 1 — Supplementary material. [file 1471-2156-14-78-S1.PDF]

Supplementary Materials for "Association Analysis of Complex Diseases Using Triads, Parent-child Dyads and Singleton Monads"

Appendix A Maximum Likelihood Estimations (MLE) and Likelihood Ratio Tests (LRT)

To simplify the notations for likelihoods 1, 3, and 5 to calculate $\log L$, let us denote

$$
\begin{aligned}
c_1 &= 4n_1 + 3n_2 + 3n_3 + 2n_4 + 2n_5 + 2n_6 + 2n_7 + n_8 + n_9, \\
c_2 &= n_2 + n_3 + 2n_4 + 2n_5 + 2n_6 + 2n_7 + 3n_8 + 3n_9 + 4n_{10}, \\
c_3 &= n_3 + n_4 + n_6 + n_8, \\
c_4 &= n_1 + n_2 + n_5, \\
k_1 &= 3m_1 + 2m_2 + 2m_3 + m_4 + m_5 + m_6, \\
k_2 &= m_2 + m_3 + m_4 + 2m_5 + 2m_6 + 3m_7, \\
k_3 &= m_2 + m_4 + m_6, \\
k_4 &= m_1 + m_3. \\
t_1 &= 2s_2 + s_1. \\
t_2 &= s_1 + 2s_0. \\
t_3 &= s_1. \\
t_4 &= s_2.
\end{aligned}
$$

Then, we may write the joint log-likelihood as follows

$$
\begin{aligned}
\log L &= \log L_{Triads} + \log L_{Parent-Child-Dyads} + \log L_{Affected-Monads} \\
&= (c_1 + k_1 + t_1)\log p + (c_2 + k_2 + t_2)\log(1-p) + (c_3 + k_3 + t_3)\log\psi_1 \\
&\quad +(c_4 + k_4 + t_4)\log\psi_2 - (n + m + s)\log R.
\end{aligned}
$$

A.1 Null Hypothesis of No Association $H_0 : \psi_1 = \psi_2 = 1$

Under $H_0 : \psi_1 = \psi_2 = 1$, we have $R = p^2\psi_2 + 2pq\psi_1 + q^2 = (p+q)^2 = 1$. Then,

$$\begin{aligned}\log L &= (c_1 + k_1 + t_1)\log p + (c_2 + k_2 + t_2)\log q \\ &= (c_1 + k_1 + t_1)\log p + (c_2 + k_2 + t_2)\log(1-p).\end{aligned}$$

This implies that

$$\begin{aligned}\frac{d\log L(p)}{dp} &= \frac{c_1 + k_1 + t_1}{p} - \frac{c_2 + k_2 + t_2}{1-p} = 0 \\ \Rightarrow \hat{p} = p_0 &= \frac{c_1 + k_1 + t_1}{c_1 + k_1 + t_1 + c_2 + k_2 + t_2}.\end{aligned}$$

A.2 Unrestricted Model $H_{Unr} : \psi_1 \geq 0, \psi_2 \geq 0$

Under $H_{Unr} : \psi_1 \geq 0, \psi_2 \geq 0$,

$$\begin{aligned}R &= p^2\psi_2 + 2pq\psi_1 + q^2 \\ &= p^2\psi_2 + 2(p - p^2)\psi_1 + q^2.\end{aligned}$$

Then, the log-likelihood is

$$\begin{aligned}\log L &= (c_1 + k_1 + t_1)\log p + (c_2 + k_2 + t_2)\log(1-p) \\ &\quad + (c_3 + k_3 + t_3)\log\psi_1 + (c_4 + k_4 + t_4)\log\psi_2 - (n + m + s)\log R.\end{aligned}$$

Hence, we have

$$\begin{aligned}\frac{d\log L(p,\psi_1,\psi_2)}{dp} &= \frac{c_1 + k_1 + t_1}{p} - \frac{c_2 + k_2 + t_2}{1-p} \\ &\quad -2(n+m+s)\frac{p\psi_2 + (1-2p)\psi_1 + p - 1}{p^2\psi_2 + (2p - 2p^2)\psi_1 + q^2}, \\ \frac{d\log L(p,\psi_1,\psi_2)}{d\psi_1} &= \frac{c_3 + k_3 + t_3}{\psi_1} - 2(n+m+s)\frac{p - p^2}{p^2\psi_2 + (2p - 2p^2)\psi_1 + q^2}, \\ \frac{d\log L(p,\psi_1,\psi_2)}{d\psi_2} &= \frac{c_4 + k_4 + t_4}{\psi_2} - (n+m+s)\frac{p^2}{p^2\psi_2 + (2p - 2p^2)\psi_1 + q^2}.\end{aligned}$$

For

$$J = \begin{bmatrix} \frac{d^2 \log L(p,\psi_1,\psi_2)}{dp^2} & \frac{d^2 \log L(p,\psi_1,\psi_2)}{dpd\psi_1} & \frac{d^2 \log L(p,\psi_1,\psi_2)}{dpd\psi_2} \\ \frac{d^2 \log L(p,\psi_1,\psi_2)}{dpd\psi_1} & \frac{d^2 \log L(p,d\psi_1,\psi_2)}{d\psi_1^2} & \frac{d^2 \log L(p,\psi_1,\psi_2)}{d\psi_1 d\psi_2} \\ \frac{d^2 \log L(p,\psi_1,\psi_2)}{dpd\psi_2} & \frac{d^2 \log L(p,\psi_1,\psi_2)}{d\psi_1 d\psi_2} & \frac{d^2 \log L(p,\psi_1,\psi_2)}{d\psi_2^2} \end{bmatrix},$$

we have

$$\begin{aligned}
\frac{d^2 \log L(p,\psi_1,\psi_2)}{dp^2} &= -\frac{c_1+k_1+t_1}{p^2} - \frac{c_2+k_2+t_2}{(1-p)^2} \\
&\quad -2(n+m+s)\left[\frac{\psi_2-2\psi_1+1}{p^2\psi_2+(2p-2p^2)\psi_1+q^2} - \frac{2(p\psi_2+(1-2p)\psi_1+p-1)^2}{(p^2\psi_2+(2p-2p^2)\psi_1+q^2)^2}\right], \\
\frac{d^2 \log L(p,\psi_1,\psi_2)}{dpd\psi_1} &= -2(n+m+s)\left[\frac{1-2p}{p^2\psi_2+(2p-2p^2)\psi_1+q^2} - \frac{2p(1-p)(p\psi_2+(1-2p)\psi_1+p-1)}{(p^2\psi_2+(2p-2p^2)\psi_1+q^2)^2}\right], \\
\frac{d^2 \log L(p,\psi_1,\psi_2)}{dpd\psi_2} &= -2(n+m+s)\left[\frac{p}{p^2\psi_2+(2p-2p^2)\psi_1+q^2} - \frac{p^2(p\psi_2+(1-2p)\psi_1+p-1)}{(p^2\psi_2+(2p-2p^2)\psi_1+q^2)^2}\right], \\
\frac{d^2 \log L(p,\psi_1,\psi_2)}{d\psi_1^2} &= -\frac{c_3+k_3+t_3}{\psi_1^2} + 4(n+m+s)\frac{p^2(1-p)^2}{(p^2\psi_2+(2p-2p^2)\psi_1+q^2)^2}, \\
\frac{d^2 \log L(p,\psi_1,\psi_2)}{d\psi_1 d\psi_2} &= 2(n+m+s)\frac{p^3(1-p)}{(p^2\psi_2+(2p-2p^2)\psi_1+q^2)^2}, \\
\frac{d^2 \log L(p,\psi_1,\psi_2)}{d\psi_2^2} &= -\frac{c_4+k_4+t_4}{\psi_2^2} + (n+m+s)\frac{p^4}{(p^2\psi_2+(2p-2p^2)\psi_1+q^2)^2}.
\end{aligned}$$

By Newton-Raphson method, we have iteration equation

$$(p^{j+1},\psi_1^{j+1},\psi_2^{j+1}) = (p^j,\psi_1^j,\psi_2^j) - J^{-1}\begin{bmatrix} \frac{d\log L(p^j,\psi_1^j,\psi_2^j)}{dp} \\ \frac{d\log L(p^j,\psi_1^j,\psi_2^j)}{d\psi_1} \\ \frac{d\log L(p^j,\psi_1^j,\psi_2^j)}{d\psi_2} \end{bmatrix}.$$

A.3 Dominant Model $H_{Dom}:\psi_1=\psi_2$

Under $H_{Dom}:\psi_1=\psi_2$, we have

$$\begin{aligned}
R &= p^2\psi_2 + 2pq\psi_1 + q^2 \\
&= p^2\psi_1 + 2p(1-p)\psi_1 + q^2 \\
&= p^2\psi_1 + 2p\psi_1 - 2p^2\psi_1 + q^2 \\
&= 2p\psi_1 - p^2\psi_1 + q^2.
\end{aligned}$$

Then, we have

$$\begin{aligned}\log L &= (c_1+k_1+t_1)\log p + (c_2+k_2+t_2)\log(1-p) \\ &\quad +(c_3+k_3+t_3+c_4+k_4+t_4)\log\psi_1 - (n+m+s)\log R.\end{aligned}$$

This implies that

$$\begin{aligned}\frac{d\log L(p,\psi_1)}{dp} &= \frac{c_1+k_1+t_1}{p} - \frac{c_2+k_2+t_2}{1-p} - 2(n+m+s)\frac{\psi_1 - p\psi_1 + p - 1}{2p\psi_1 - p^2\psi_1 + q^2}, \\ \frac{d\log L(p,\psi_1)}{d\psi_1} &= \frac{c_3+k_3+t_3+c_4+k_4+t_4}{\psi_1} - (n+m+s)\frac{2p-p^2}{2p\psi_1 - p^2\psi_1 + q^2}.\end{aligned}$$

For

$$J = \begin{bmatrix} \frac{d^2\log L(p,\psi_1)}{dp^2} & \frac{d^2\log L(p,\psi_1)}{dpd\psi_1} \\ \frac{d^2\log L(p,\psi_1)}{dpd\psi_1} & \frac{d^2\log L(p,\psi_1)}{d\psi_1^2} \end{bmatrix},$$

we have

$$\begin{aligned}\frac{d^2\log L(p,\psi_1)}{dp^2} &= -\frac{c_1+k_1+t_1}{p^2} - \frac{c_2+k_2+t_2}{(1-p)^2} \\ &\quad -2(n+m+s)\left[\frac{1-\psi_1}{2p\psi_1 - p^2\psi_1 + q^2} - \frac{2(\psi_1 - p\psi_1 + p - 1)^2}{(2p\psi_1 - p^2\psi_1 + q^2)^2}\right], \\ \frac{d^2\log L(p,\psi_1)}{dpd\psi_1} &= -2(n+m+s)\left[\frac{1-p}{2p\psi_1 - p^2\psi_1 + q^2} - \frac{(\psi_1 - p\psi_1 + p - 1)(2p-p^2)}{(2p\psi_1 - p^2\psi_1 + q^2)^2}\right], \\ \frac{d^2\log L(p,\psi_1)}{d\psi_1^2} &= -\frac{c_3+k_3+t_3+c_4+k_4+t_4}{\psi_1^2} + (n+m+s)\frac{(2p-p^2)^2}{(2p\psi_1 - p^2\psi_1 + q^2)^2}.\end{aligned}$$

By Newton-Raphson method, we have iteration equation

$$(p^{j+1}, \psi_1^{j+1}) = (p^j, \psi_1^j) - J^{-1}\begin{bmatrix} \frac{d\log L(p^j,\psi_1^j)}{dp} \\ \frac{d\log L(p^j,\psi_1^j)}{d\psi_1} \end{bmatrix}.$$

A.4 Recessive $H_{Rec}: \psi_1 = 1$

Under $H_{Rec}: \psi_1 = 1$, we have

$$\begin{aligned}R &= p^2\psi_2 + 2pq\psi_1 + q^2 \\ &= p^2\psi_2 - p^2 + 1.\end{aligned}$$

Then, $\log L = (c_1+k_1+t_1)\log p + (c_2+k_2+t_2)\log(1-p) + (c_4+k_4+t_4)\psi_2 - (n+m+s)\log R$. Hence, we have

$$\begin{aligned}
\frac{d\log L(p,\psi_2)}{dp} &= \frac{c_1+k_1+t_1}{p} - \frac{c_2+k_2+t_2}{1-p} - 2(n+m+s)\frac{p(\psi_2-1)}{p^2\psi_2-p^2+1},\\
\frac{d\log L(p,\psi_2)}{d\psi_2} &= \frac{c_4+k_4+t_4}{\psi_2} - (n+m+s)\frac{p^2}{p^2\psi_2-p^2+1}.
\end{aligned}$$

For

$$J = \begin{bmatrix} \frac{d^2\log L(p,\psi_2)}{dp^2} & \frac{d^2\log L(p,\psi_2)}{dpd\psi_2} \\ \frac{d^2\log L(p,\psi_2)}{dpd\psi_2} & \frac{d^2\log L(p,\psi_2)}{d\psi_2^2} \end{bmatrix},$$

we have

$$\begin{aligned}
\frac{d^2\log L(p,\psi_2)}{dp^2} &= -\frac{c_1+k_1+t_1}{p^2} - \frac{c_2+k_2+t_2}{(1-p)^2}\\
&\quad -2(n+m+s)\left[\frac{\psi_2-1}{p^2\psi_2-p^2+1} - \frac{2p^2(\psi_2-1)^2}{(p^2\psi_2-p^2+1)^2}\right],\\
\frac{d^2\log L(p,\psi_2)}{dpd\psi_2} &= -2(n+m+s)\left[\frac{p}{p^2\psi_2-p^2+1} - \frac{p^3(\psi_2-1)}{(p^2\psi_2-p^2+1)^2}\right],\\
\frac{d^2\log L(p,\psi_2)}{d\psi_2^2} &= -\frac{c_4+k_4+t_4}{\psi_2^2} + (n+m+s)\frac{p^4}{(p^2\psi_2-p^2+1)^2}.
\end{aligned}$$

By Newton-Raphson method, we have the following iteration equation

$$(p^{j+1},\psi_2^{j+1}) = (p^j,\psi_2^j) - J^{-1}\begin{bmatrix} \frac{d\log L(p^j,\psi_2^j)}{dp} \\ \frac{d\log L(p^j,\psi_2^j)}{d\psi_2} \end{bmatrix}.$$

A.5 Multiplicative Model $H_{Mult}:\psi_2=\psi_1^2$

Under $H_{Mult}:\psi_2=\psi_1^2$, we have

$$R = p^2\psi_2 + 2pq\psi_1 + q^2 = p^2\psi_1^2 + 2pq\psi_1 + q^2 = (p\psi_1+q)^2 = (p\psi_1+1-p)^2$$

Then,

$$\begin{aligned}
\log L &= (c_1+k_1+t_1)\log p + (c_2+k_2+t_2)\log(1-p)\\
&\quad +(c_3+k_3+t_3)\log\psi_1 + (c_4+k_4+t_4)\log\psi_1^2 - (n+m+s)\log R\\
&= (c_1+k_1+t_1)\log p + (c_2+k_2+t_2)\log(1-p)\\
&\quad +(c_3+k_3+t_3+2c_4+2k_4+2t_4)\log\psi_1 - 2(n+m+s)\log(p\psi_1+1-p).
\end{aligned}$$

Hence, one has

$$\begin{aligned}\frac{d\log L(p,\psi_1)}{dp} &= \frac{c_1+k_1+t_1}{p}-\frac{c_2+k_2+t_2}{1-p}-2(n+m+s)\frac{\psi_1-1}{p\psi_1+1-p},\\ \frac{d\log L(p,\psi_1)}{d\psi_1} &= \frac{c_3+k_3+t_3+2c_4+2k_4+2t_4}{\psi_1}-2(n+m+s)\frac{p}{p\psi_1+1-p}.\end{aligned}$$

For

$$J=\left[\begin{array}{cc}\frac{d^2\log L(p,\psi_1)}{dp^2} & \frac{d^2\log L(p,\psi_1)}{dpd\psi_1}\\ \frac{d^2\log L(p,\psi_1)}{dpd\psi_1} & \frac{d^2\log L(p,\psi_1)}{d\psi_1^2}\end{array}\right],$$

we have

$$\begin{aligned}\frac{d^2\log L(p,\psi_1)}{dp^2} &= -\frac{c_1+k_1+t_1}{p^2}-\frac{c_2+k_2+t_2}{(1-p)^2}+2(n+m+s)\frac{(\psi_1-1)^2}{(p\psi_1+1-p)^2},\\ \frac{d^2\log L(p,\psi_1)}{dpd\psi_1} &= -2(n+m+s)\frac{1}{(p\psi_1+1-p)^2},\\ \frac{d^2\log L(p,\psi_1)}{d\psi_1^2} &= -\frac{c_3+k_3+t_3+2c_4+2k_4+2t_4}{\psi_1^2}+2(n+m+s)\frac{p^2}{(p\psi_1+1-p)^2}.\end{aligned}$$

By Newton-Raphson method, we have iteration equation

$$(p^{j+1},\psi_1^{j+1})=(p^j,\psi_1^j)-J^{-1}\left[\begin{array}{c}\frac{d\log L(p^j,\psi_1^j)}{dp}\\ \frac{d\log L(p^j,\psi_1^j)}{d\psi_1}\end{array}\right].$$

A.6 Additive Model $H_{Add}:\psi_2=2\psi_1-1$

Under $H_{Add}:\psi_2=2\psi_1-1$, we have

$$\begin{aligned}R &= p^2\psi_2+2pq\psi_1+q^2\\ &= 2p^2\psi_1+2p(1-p)\psi_1+q^2-p^2\\ &= 2p^2\psi_1+2p\psi_1-2p^2\psi_1+q-p\\ &= 2p\psi_1+1-2p.\end{aligned}$$

Then, we have

$$\begin{aligned}\log L &= (c_1+k_1+t_1)\log p+(c_2+k_2+t_2)\log(1-p)\\ &\quad +(c_3+k_3+t_3)\log\psi_1+(c_4+k_4+t_4)\log(2\psi_1-1)-(n+m+s)\log R.\end{aligned}$$

This implies that

$$\frac{d\log L(p,\psi_1)}{dp} = \frac{c_1+k_1+t_1}{p} - \frac{c_2+k_2+t_2}{1-p} - 2(n+m+s)\frac{\psi_1-1}{2p\psi_1+1-2p},$$
$$\frac{d\log L(p,\psi_1)}{d\psi_1} = \frac{c_3+k_3+t_3}{\psi_1} + \frac{2(c_4+k_4+t_4)}{2\psi_1-1} - 2(n+m+s)\frac{p}{2p\psi_1+1-2p}.$$

For

$$J = \begin{bmatrix} \frac{d^2\log L(p,\psi_1)}{dp^2} & \frac{d^2\log L(p,\psi_1)}{dpd\psi_1} \\ \frac{d^2\log L(p,\psi_1)}{dpd\psi_1} & \frac{d^2\log L(p,\psi_1)}{d\psi_1^2} \end{bmatrix},$$

we have

$$\frac{d^2\log L(p,\psi_1)}{dp^2} = -\frac{c_1+k_1+t_1}{p^2} - \frac{c_2+k_2+t_2}{(1-p)^2} + 4(n+m+s)\frac{(\psi_1-1)^2}{(2p\psi_1+1-2p)^2},$$
$$\frac{d^2\log L(p,\psi_1)}{dpd\psi_1} = -2(n+m+s)\frac{1}{(2p\psi_1+1-2p)^2},$$
$$\frac{d^2\log L(p,\psi_1)}{d\psi_1^2} = -\frac{c_3+k_3+t_3}{\psi_1^2} - \frac{4(c_4+k_4+t_4)}{(2\psi_1-1)^2} + 4(n+m+s)\frac{p^2}{(2p\psi_1+1-2p)^2}.$$

By Newton-Raphson method, we have iteration equation

$$(p^{j+1},\psi_1^{j+1}) = (p^j,\psi_1^j) - J^{-1}\begin{bmatrix} \frac{d\log L(p^j,\psi_1^j)}{dp} \\ \frac{d\log L(p^j,\psi_1^j)}{d\psi_1} \end{bmatrix}.$$
